# Reorganization of Visual Callosal Connections Following Alterations of Retinal Input and Brain Damage

**DOI:** 10.3389/fnsys.2016.00086

**Published:** 2016-11-14

**Authors:** Laura Restani, Matteo Caleo

**Affiliations:** Neuroscience Institute, National Research Council (CNR)Pisa, Italy

**Keywords:** corpus callosum, visual system, retinal input, callosal plasticity, splenium, transcallosal inhibition, cortical lesion, visual cortex plasticity

## Abstract

Vision is a very important sensory modality in humans. Visual disorders are numerous and arising from diverse and complex causes. Deficits in visual function are highly disabling from a social point of view and in addition cause a considerable economic burden. For all these reasons there is an intense effort by the scientific community to gather knowledge on visual deficit mechanisms and to find possible new strategies for recovery and treatment. In this review, we focus on an important and sometimes neglected player of the visual function, the corpus callosum (CC). The CC is the major white matter structure in the brain and is involved in information processing between the two hemispheres. In particular, visual callosal connections interconnect homologous areas of visual cortices, binding together the two halves of the visual field. This interhemispheric communication plays a significant role in visual cortical output. Here, we will first review the essential literature on the physiology of the callosal connections in normal vision. The available data support the view that the callosum contributes to both excitation and inhibition to the target hemisphere, with a dynamic adaptation to the strength of the incoming visual input. Next, we will focus on data showing how callosal connections may sense visual alterations and respond to the classical paradigm for the study of visual plasticity, i.e., monocular deprivation (MD). This is a prototypical example of a model for the study of callosal plasticity in pathological conditions (e.g., strabismus and amblyopia) characterized by unbalanced input from the two eyes. We will also discuss the findings of callosal alterations in blind subjects. Noteworthy, we will discuss data showing that inter-hemispheric transfer mediates recovery of visual responsiveness following cortical damage. Finally, we will provide an overview of how callosal projections dysfunction could contribute to pathologies such as neglect and occipital epilepsy. A particular focus will be on reviewing noninvasive brain stimulation techniques and optogenetic approaches that allow to selectively manipulate callosal function and to probe its involvement in cortical processing and plasticity. Overall, the data indicate that experience can potently impact on transcallosal connectivity, and that the callosum itself is crucial for plasticity and recovery in various disorders of the visual pathway.

## Introduction

The visual system is one of the most popular topics of investigation in physiology, since the classical study of Hubel and Wiesel in the sixties. As a consequence, a huge amount of literature has been generated, with reports spanning from basic research to clinical studies focusing either on the physiology of the visual system or possible recovery from pathologies or visual deficits (Hensch, 2005; Morishita and Hensch, 2008; Baroncelli et al., 2011). The popularity of the visual system arises from the accessibility of its structures and easy technical manipulation, compared to other brain areas, but also from the importance of vision for human behavior. Indeed visual disorders have a profound impact on single subjects and human society.

Currently, several efficient and non-invasive techniques are available to study visual physiology in humans. However, numerous studies on mechanisms of visual system physiology are still carried out in animal models. In the last decade, numerous articles have taken advantage of optical manipulation to dissect sensory system circuitry (Petreanu et al., 2007; Carter and de Lecea, 2011; Cardin, 2012; Gaub et al., 2015). However, the majority of these studies focused the attention on alterations or activity of the major players of the visual system, such as retina, thalamus and visual cortex, neglecting the corpus callosum (CC).

The CC is not just a mere connection channel for visual information but is also an important player in the construction of a coherent visual percept. Given its primary role of “connection”, it is often neglected by authors and considered as only as a passive highway of visual information through visual neuronal areas. The CC plays a critical role not only in basic physiology of the visual system, but it is fundamental in plasticity phenomena, as recently emerged (Caleo et al., 2007; Restani et al., 2009; Cerri et al., 2010; Pietrasanta et al., 2012, 2014). Indeed plastic rearrangements are induced in visual callosal connections as a consequence of visual system deficits, such as alteration of retinal inputs and brain damage.

## The Corpus Callosum: Anatomical and Physiological Framework

The CC is the largest white matter structure in the brain and in humans contain more than 200 million fibers. Callosal connections interlink homologous and non-homologous cortical areas situated in the two cerebral hemispheres (Lewis and Olavarria, 1995; Funnell et al., 2000; Houzel et al., 2002; Bocci et al., 2014).

Nature and function of the CC are of interest, as it connects the most important areas of the neocortex. Therefore, alterations in its structure do not only impact on visual perception, but may also affect cognitive functions and result in developmental disorders. Abnormalities in size and structure of the callosum have been found in patients with schizophrenia, autism, mental retardation, Down’s syndrome, developmental languages disorders (Hynd et al., 1995; Nagy et al., 2004; van der Knaap and van der Ham, 2011), suggesting that callosal alterations might contribute to pathological features, even if a causal relationship is difficult to demonstrate.

Despite the amount of work devoted to the study of physiology and pathology of CC especially in the 1980s, we still do not hold a complete understanding of the nature and physiology of interhemispheric integration, especially of how CC dysfunction could contribute to several central nervous system (CNS) pathologies. In this view, the visual system offers an excellent possibility to analyze consequences of callosal connections alterations following input alteration or plasticity. Classical plasticity models in rodent visual cortex allow the study of interactions between CC and other brain areas and to correlate functional changes in interhemispheric connectivity to specific dysfunctions of the CNS.

Visual callosal connections mature late in humans (until adolescence), until around 1 month in cats and around postnatal day 15 (P15) in rodents (Nagy et al., 2004; Mizuno et al., 2007; Pietrasanta et al., 2012; Westerhausen et al., 2016). First, the CC enlarges caudally and then develops rostrally (Hynd et al., 1995). Similarly, myelination occurs slowly and with a caudal—rostral development (Hynd et al., 1995; Nuñez et al., 2000; Doron and Gazzaniga, 2008; Markham et al., 2009; Bercury and Macklin, 2015).

In primary sensory areas, interhemispheric projections link essentially homotopic zones. In all mammals, each hemisphere receives information from the opposite visual hemifield. In cats and primates, with a large binocular visual field, only axons arising from the nasal half of the retina cross, while in rodents, with more lateral eyes and limited binocular vision, a higher percentage of fibers crosses (Houzel and Milleret, 1999). Thus, the visual world is discontinuously represented as seen in cortical maps, split along the central vertical meridian. Despite this segmentation, we have perception of continuity and this is at a high degree because of the CC. Indeed, one of the accepted role of the CC and its essential functions is to guarantee the continuity of sensory maps across the hemispheres. This fusion is achieved by precise, reciprocal, point-to-point callosal connections between cortical neurons, whose receptive fields are located along the vertical meridian.

The basic layout of the callosal connections linking primary visual cortex (V1) has been investigated mostly in cats, by anatomical and electrophysiological techniques. Callosal connections form a dense stripe along the border of areas 17 and 18 (i.e., primary and secondary visual cortex Jacobson and Trojanowski, 1974; Blakemore et al., 1983; Olavarria and van Sluyters, 1995; Houzel and Milleret, 1999). Also in other species, from rats to macaques to humans, a similar organization has been reported (Jacobson and Trojanowski, 1974; Cusick and Lund, 1982; Van Essen et al., 1982; Clarke and Miklossy, 1990; Mizuno et al., 2007). More recent studies with diffusion tensor imaging (DTI) and fiber tracking have provided a detailed description of callosal connections in human V1 (Dougherty et al., 2005; Putnam et al., 2010; Saenz and Fine, 2010). In particular, a significant asymmetry in callosal anatomy has been found, with more connections from the right to the left hemisphere than vice versa (Putnam et al., 2010). In cats, analysis of single callosal axons has demonstrated that some branches terminate within the core of area 17 (in addition to the dense terminations at the 17/18 border). Similar callosal projections to the core of area 17 have been detected in humans (Putnam et al., 2010). These branches might provide mostly subthreshold activation of the cortical neurons (Houzel et al., 2002). Callosal arborizations form synaptic boutons mainly in supragranular layers and layer V (Mizuno et al., 2007; Rochefort et al., 2009).

In animal models, a series of physiological studies have been performed, showing that the 17/18 border is a transition zone: neurons in this boundary have receptive fields mapping in the vertical midline, and others have receptive fields mapping a small region in the ipsilateral hemifield (Blakemore et al., 1983; Payne, 1990, 1994; Payne and Siwek, 1991; White et al., 1999). Split-chiasm experiments revealed that transcallosal and ipsilateral, geniculocortical inputs, converging onto a given target neuron, are precisely matched so that receptive fields plotted through both pathways are virtually superimposed (Berlucchi and Rizzolatti, 1968; Milleret, 1994). Combining retrograde tracing with latex microspheres and 2-deoxyglucose (2-DG) autoradiography, Schmidt et al. (1997) demonstrated that callosal neurons preferentially link iso-oriented columns in the two hemispheres in cats. This specific organization of interhemispheric axons linking cortical regions representing the same orientation was confirmed by Rochefort et al. (2009), combining *in vivo* optical imaging of intrinsic signals with labeling of callosal axons. The topographical selectivity was confirmed by functional experiments performed in ferrets, demonstrating that neurons responding to the same stimulus orientation are interconnected. On the contrary, inhibition seems to occur both between iso-oriented and non-iso-oriented neurons (Makarov et al., 2008). Combining reversible thermal deactivation of one hemisphere and electrophysiology/imaging of intrinsic signals, transcallosal modulation of response properties of cortical neurons was studied in ferrets and cats. The authors found that callosal input influences on both the strength and specificity of the responses to stimulus orientation and direction of motion (Schmidt et al., 2010). These data are consistent with the view that inter-hemispheric connections contribute to a unified perception of the visual scene. In particular, a moving object in the visual space activates transcallosal connections which could “pre-alert” neurons in the contralateral hemisphere, decreasing the threshold for firing and preparing the network to process a stimulus that crosses the vertical meridian (Houzel et al., 2002). This intriguing hypothesis was tested by Peiker et al. (2013): they found that neuronal responses to a movement away from the deactivated hemifield were more strongly reduced in absence of callosal input than responses to the counter-movement. This experimental result supports a role for callosal inputs in the processing of motion directions. A callosally-mediated anticipatory activity may permit more accurate and faithful processing of stimuli that move across the vertical meridian (Houzel et al., 2002; Peiker et al., 2013).

Callosal connections display also specific features regarding spatial frequency. Ribot et al. (2013) investigated the spatial frequency organization at the boundary between area 17/18, where callosal axons terminate densely. Using optical imaging techniques, they recorded intrinsic signals at different spatial frequencies, finding that in the transition zone the topographic organization of spatial frequency is dependent similarly on both the geniculo-cortical output and the callosal connections (Ribot et al., 2013).

Recently, Olavarria’s group published data demonstrating, in rats, eye-specific domains in the binocular portion of V1 (Laing et al., 2015). Transcallosal connections appear to colocalize primarily with ipsilateral eye domains in the binocular region of V1, similar to the organization previously reported in the cat (Olavarria, 2001). This anatomical arrangement suggest a potential contribution of callosal connections to binocularity in rodent visual cortex (Restani et al., 2009; Cerri et al., 2010). In particular, our group recorded binocularity of cells in one hemisphere of young rats. To dissect the role of callosal input in binocularity, we silenced interhemispheric connections by intracortical injection of muscimol, an agonist of GABA_A_ receptors. We recorded spiking activity and we found that responses driven from the ipsilateral eye were dampened, while contralateral eye inputs were basically unaffected (Restani et al., 2009). We also analyzed the contralateral-to-ipsilateral (C/I) visual evoked potential (VEP) ratio (Pietrasanta et al., 2014). We reported that silencing of callosal communication resulted in a robust shift in eye preference in favor of the contralateral eye, increasing the C/I ratio. In a complementary experiment, we recorded VEP before and after elimination of thalamic input via stereotaxic tetrodotoxin (TTX) injection into the geniculate. We found that TTX silencing produced a strong decrease of C/I VEP ratio, towards the ipsilateral eye. The reduction of C/I ratio was, in particular, due to a reduction of contralateral eye responses, while ipsilateral eye responses were reduced much less (Cerri et al., 2010). Overall, these data demonstrate that callosal input carries excitatory drive in young animals to the opposite cortex, and this input comes mainly from the ipsilateral eye. Studies in mice have confirmed the key role for the callosum in providing ipsilateral eye inputs to cortical neurons, using inactivation of one hemisphere and either patch-clamp recordings of individual cells (Zhao et al., 2013) or imaging of intrinsic signals (Dehmel and Löwel, 2014).

**Figure 1:**
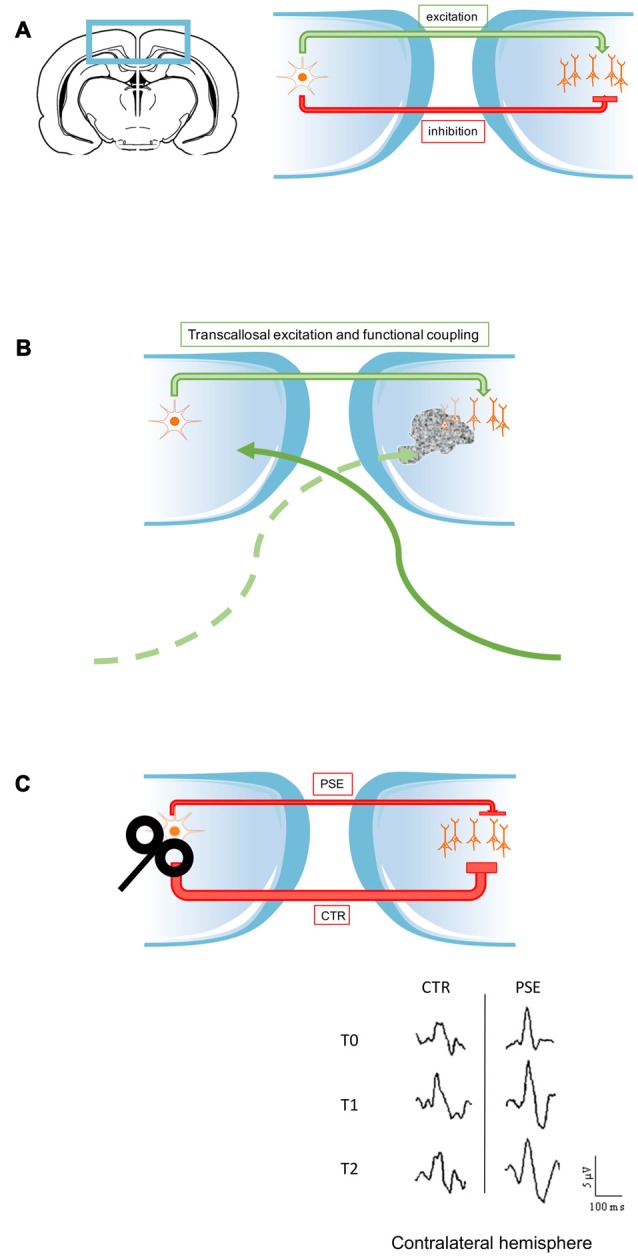
**Schematic illustration of the role of interhemispheric input in visual physiology and pathology. (A)** In physiological conditions the corpus callosum (CC) provides both excitatory and inhibitory input to the contralateral hemisphere (see first section of this review). **(B)** Callosal communication is at the basis of the functional coupling of the two hemispheres, but it could also contribute to provide excitatory input to spared cortical regions, when lesions occurred in the opposite side (Kiper et al., 2002; Knyazeva et al., 2002; Kavcic et al., 2015). **(C)** In some pathological conditions, the transcallosal pathway could be impaired and transfer abnormal inhibitory input. In photosensitive patients (PSE), following low frequency repetitive transcranial magnetic stimulation (rTMS) in one side, the untreated side displayed a persistent enhancement of visual evoked potentials (VEPs) amplitude (T2). This suggests that less effective inhibition provided by callosal projections might be at the basis of the prolonged increase of visual responses measured in the untreated side. This may potentially contribute to the pathophysiology of PSE (Bocci et al., 2016).

It is well established that the CC provides both excitatory and inhibitory input to visual cortex (Makarov et al., 2008; for specific reviews, see Bloom and Hynd, 2005; Bocci et al., 2014; see also Figure 1A). For example, cooling or GABA injections in one hemisphere decrease cell responsiveness in a subset of contralateral neurons, suggesting transcallosal excitatory drive to these neurons (Payne et al., 1991; Sun et al., 1994). Transcallosal influences are different in specific cortical layers. More than 90% of neurons in layers II/III and V/VI were influenced by cooling of one hemisphere, while only half of recorded cells in layer IV displayed changes in responsiveness after callosal inactivation (Payne, 1994). Schmidt’s group (Wunderle et al., 2013) quantified firing rates of neurons before and after thermal deactivation of the opposite hemisphere, and observed a mixture of excitatory and inhibitory transcallosal effects in cat visual cortex. In particular, stimulation with high-contrast, full-field gratings revealed both excitatory and inhibitory transcallosal actions, while almost exclusively facilitating effects were detected with a less salient and unstructured stimulus (random dot textures). Thus, significant inhibitory actions via the callosum only come into action when the network is driven by a strong feed forward drive. These data indicate that the nature of callosal effects is not fixed but rather dynamically adapted to the incoming visual stimuli (Wunderle et al., 2013).

Following callosal inactivation, a subset of neurons increase their response, compatible with the removal of a callosally driven inhibition (Payne et al., 1991; Payne, 1994; Sun et al., 1994; Bocci et al., 2011). In one experiment on human subjects, visual evoked potentials triggered by grating stimuli of different contrasts were recorded before and after functional inactivation of the occipital cortex of one hemisphere, via low-frequency repetitive transcranial magnetic stimulation (rTMS; Bocci et al., 2011). The authors found that during inhibition of transcallosal pathway induced by rTMS, neural responses in the contralateral side increased, specifically for mid- high-contrast stimuli. These data are in support of an overall inhibitory function of transcallosal communication between visual cortices at least at mid-high contrasts in humans (Bocci et al., 2011). On the other hand, Schmidt’s group (Wunderle et al., 2015) reported in cats a clear evidence for contrast gain modulation by callosal network. Callosal input was able to increase firing rate of target neurons at mid-high contrast, but was ineffective at low contrast (Berardi et al., 1987; Wunderle et al., 2015). Wunderle et al. (2015) found that manipulation of callosal input can modulate contrast gain in the target hemisphere via either a change in the semisaturation contrast of a neuron’s contrast response function, or via a change in the maximal response of a cell (i.e., shifting the contrast response curve vertically). One hypothesis is that the relative contribution of direct interhemispheric and indirect intrinsic inputs determines the scaling mechanism for each particular neuron in the target hemisphere (Wunderle et al., 2015).

The notion of a mixture of excitation and inhibition carried by transcallosal pathways is consistent with anatomical data. Anatomically, a high percentage of callosal neurons are large pyramidal cells, but callosal neurons do not constitute a homogenous population, since they have different morpho-chemical phenotypes. Among those are spiny stellate, but also smooth stellate and fusiform cells, which suggests that at least some callosal neurons could use inhibitory transmitters (Buhl and Singer, 1989). This is well-matched with the occasional observation of symmetric callosal synapses as well as with the electrophysiological disclosure of short-latency transcallosal inhibition (Payne and Siwek, 1991; Makarov et al., 2008). Our group performed retrograde tracing combined GABA immunostaining in rat visual cortex and found that the percentage of inhibitory callosal units was quite low (1%, Restani et al., 2009). On the other hand, it is well established that callosal neurons are able to recruit indirectly inhibitory interneurons (specifically, parvalbumin (PV)-positive cells) on the contralateral side (Toyama et al., 1974; Innocenti, 1980; Martin et al., 1983; Restani et al., 2009). Indeed electron microscopy has demonstrated that PV-labeled profiles and unlabeled dendritic spines of deep cortical layer neurons receive synapses from the contralateral hemisphere (Karayannis et al., 2007). The authors performed stimulation of callosal fibers and were able to record monosynaptic excitatory postsynaptic currents in both layer VI pyramidal neurons and GABAergic interneurons, immunopositive for PV (Karayannis et al., 2007).

## Callosal Plasticity: How Visual Input Impacts on Interhemispheric Connections

One of the strongest pillars of the theory of neuronal plasticity is that retinal input influences development and organization of the principal stations of the visual system, such as dorsal lateral geniculate nucleus (dLGN) and visual cortex. A huge effort has been dedicated to investigate this field. It is for example broadly accepted that retinal waves drive segregation of axons in the dLGN (Wong, 1999; Stellwagen and Shatz, 2002; Torborg et al., 2005) and that monocular deprivation (MD) influences the size of ocular dominance (OD) columns and OD distribution in the V1 of monkeys, cats and rodents (Wiesel and Hubel, 1963, 1965; LeVay et al., 1980; Maffei et al., 1992; Horton and Hocking, 1997).

Here we would like to focus our interest on the effects of classical retinal manipulations (enucleation, MD, strabismus, blindness) on anatomy and physiology of the CC. Indeed, while the influence of retinal drive on dLGN and visual cortex has been extensively studied, much less is known on its consequences on functions and structure of interhemispheric connections.

The majority of the studies on callosal reorganization have been conducted in animal models. As mentioned in the first section above, callosal neurons connect retinotopically corresponding loci. As for many other structures in brain, electrical activity during development is the main sculptor of neuronal circuits. The CC is also subjected to this remodeling process, because callosal axons are initially exuberant, but during normal development there is a partial elimination of callosal axon terminals mostly depending on activity (Innocenti and Caminiti, 1980; Innocenti, 1981; Innocenti and Price, 2005; Mizuno et al., 2007). A number of research articles focused on this issue and analyzed how retinal input could modify callosal maps. A summary of the main findings is reported in Table 1.

**Table 1:** **Summary of the main results described in the section regarding the impact of retinal activity on callosal projections**.

Experimental condition	Species	Main findings	References
Neonatal eye enucleation	Rats	Expanded callosal pathway to contralateral V1	Cusick and Lund (1982)

Neonatal eye enucleation	Rats	A dense band of callosal connections running rostrocaudally into the center of V1, in the hemisphere ipsilateral to the spared eye Alteration of the callosal map if enucleation was performed between birth and 5 days of age	Olavarria et al. (1987)

Neonatal bilateral enucleation	Rats, cats	Disrupted organization of the interhemispheric asymmetric pattern; Lateral portion of V1 receiving callosal input showed an organization similar to the medial portion, connecting mirror-symmetric loci	Olavarria and Li (1995); Olavarria and van Sluyters (1995)

Neonatal enucleation	Rats	Decreased proportion of callosal boutons making multiple postsynaptic contacts	Sorensen et al. (2003)

Enucleation	Rats, mice	Precise time window, P4–P6, during which development of retinotopical loci of callosal fibers depend on eye presence	Olavarria and Hiroi (2003)

Enucleation	Rats	Faster kinetics of NMDAR-EPSCs (if enucleation is performed between P4 and P6)	Olavarria et al. (2007)

Enucleation	Rats	Callosally evoked responses were larger than normal High density of callosal terminals in layers II and III	Toldi et al. (1989)

TTX eye	Rabbits	Widespread callosal zone, extending for one-third more into area 17 (TTX starting on postnatal day 6–7, for 3 weeks)	Grigonis and Murphy (1991)

TTX eye	Rats	No effects on callosal with prolonged TTX for two postnatal weeks	Chang et al. (1995)
Eyelid suture (bilateral)	Cats	A reduction of callosal cell number; Extension of callosal zone into contralateral visual cortex	Innocenti and Frost (1979); Innocenti and Caminiti (1980); Innocenti (1981)
Eyelid suture (monocular deprivation)	Cats	Variability in the organization of ocular dominance columns within the callosal zone; Ectopic callosal cells (probably due to an impaired elimination)	Alekseenko et al. (2009)

Eyelid suture (adult)	Cats	Basically normal anatomy of callosal projections (the only detectable change was a wider callosal visual field towards the ipsilateral hemifield); Functionally, abnormally large receptive fields and a loss of orientation selectivity	Watroba et al. (2001)

Eyelid suture (monocular deprivation)	Rats	Functional callosal inhibition of input coming from deprived eye; Continuous silencing of callosal input throughout MD period prevented the loss of responsiveness of the deprived eye and reduced ocular dominance shift	Restani et al. (2009)

Strabismus	Cats	Enlargement of callosal zone, with somas of callosal neurons occupying a wider portion of visual cortex	Elberger et al. (1983)

Strabismus	Cats	Wider portion of the callosal zone Decreased binocularity of callosal neurons	Berman and Payne (1983); Milleret and Houzel (2001); Alekseenko et al. (2006); Alekseenko et al. (2009)
Strabismus	Monkey, cats	Some units became responsive to stimuli when presented in the ipsilateral hemifield (although these units were not selective for orientation or motion)	Sugita (1996); Milleret and Houzel (2001); Watroba et al. (2001)
Strabismus	Cats	Callosal connections link predominantly territories that share the same ocular dominance	Schmidt et al. (1997)
Strabismus	Cats	Anatomical study, extension of interhemispheric terminals into the hemisphere ipsilateral to the deviated eye	Bui Quoc et al. (2012)
Blindness	Humans	Volume reduction in primary and secondary visual cortices in early onset subjects	Leporé et al. (2010)
Blindness, Anophthalmy	Humans	No spline volume differences between early blind and in anophthalmic subjects	Bock et al. (2013)
Retinoblastoma	Humans	Patients with unilateral tumors, compared to whose displaying bilateral damage, had greater fractional anisotropy, and lower diffusion (suggesting changes in myelination)	Barb et al. (2011)

### Eye Enucleation and Intravitreal Tetrodotoxin (TTX) Injection

One experimental protocol implemented eye enucleation in animals to test the impact on callosal projections.

Cusick and Lund (1982) showed how the detailed distribution of the visual callosal projection within area 17 was affected by retinogeniculocortical input to each hemisphere, finding that monocular enucleation at birth produced an expanded callosal pathway to contralateral V1, similarly to what happens following unilateral thalamic lesions performed at birth. However, in the latter case the establishment of an abnormal projection was restricted mainly to layers IV and III. The majority of anatomical studies on reorganization of transcallosal fibers after enucleation was made starting from the eighties by Olavarria and co-authors (Olavarria et al., 1987). Olavarria et al. (1987) analyzed in detail the effects of monocular or binocular eye enucleation performed at birth on development of callosal fibers in rats. They found that although the main features of callosal pattern are maintained in both groups, with a dense accumulation of callosal terminals at the border of area 17/18, there were some abnormalities: in monocularly enucleated rats, in the hemisphere ipsilateral to the spared eye, they discovered a dense band of callosal connections running rostrocaudally into the center of V1. They continued studying whether there was a critical time window, during which eye removal could alter the normal callosal fibers’ development. They discovered that enucleation allows normal development if performed at 6 days or later, but it was able to alter the callosal map if performed between birth and 5 days of age (Olavarria et al., 1987). Some years later, they deepened into the issue with an additional investigation. First, they demonstrated that callosal terminals arising from medial region of V1 link mirror-symmetric loci, whereas axons originating from more lateral border between V1/V2 came from cells located medial to the border, thus interconnecting non-mirror loci (Lewis and Olavarria, 1995). Then, they investigated if this asymmetric pattern was influenced by eye inputs. The authors looked at callosal fibers’ development following neonatal bilateral enucleation in rats and they found a disrupted organization of the interhemispheric asymmetric pattern. Using fluorescent tracers, they found that lateral V1 receiving callosal input showed an organization similar to the medial portion, connecting mirror-symmetric loci. Notably, the medial region was unaffected (Olavarria and Li, 1995). Similar results were obtained in cats (Olavarria and van Sluyters, 1995).

The role of eye presence in determining this asymmetric array of callosal terminals was further investigated at different postnatal ages. Enucleation in rats and mice was effective in changing fine callosal topography if performed between P4 and P6 (Olavarria and Hiroi, 2003). In ferrets, a similar critical period has been found: bilateral enucleation at P7 impaired normal topography of callosal map, while was not effective if performed at P20 (Bock et al., 2012). In rodents, enucleation at P4 produces anatomical data similar to those obtained with neonatal enucleation. These findings support the idea that the eyes do not exert an effect on fine callosal topography until P4. On the other hand, after P6, enucleation is ineffective on callosal development. In conclusion, authors find a very precise time window, P4–P6, during which development of retinotopical loci of callosal fibers depend on eye presence (Olavarria and Hiroi, 2003).

It is also important to mention that development of callosal connections in rodents terminates around P12/P15, thus the influence of eye presence occurs in a very early period of callosal development. Sorensen et al. (2003) published a study in which they analyzed synaptic alterations induced by neonatal enucleation, although in this case the analyses were performed when rats reached adult age. Thus, in this study they allowed animals to grow up for a longer period, during which additional plastic phenomena could occur. Nevertheless, they reported a decreased proportion of callosal boutons making multiple postsynaptic contacts.

These findings must be taken in to account especially for functional implications. As we mentioned before, callosal fibers could monosynaptically contact excitatory neurons and inhibitory cells via disynaptic contacts. Changes in callosal postsynaptic contacts could thus impact both interhemispheric balance and intracortical excitability.

Olavarria and collaborators (Olavarria et al., 2007) went on to characterize the possible effectors involved in the effects of eye enucleation on callosal topography. Intrigued by the critical and brief time window between P4 and P6, they explored this issue at molecular level, studying whether there was a correlation of this critical period with the age-related changes in the kinetics of synaptic responses mediated by the N-methyl-D-aspartate subclass of glutamate receptors (NMDARs; Olavarria et al., 2007). NMDAR are well known to play a critical role in plasticity. Authors analyzed the decay time constant of NMDAR-EPSCs recorded from slices, in which callosal neurons were retrogradely-labeled, and found that, in normal rats, the decay time constant of NMDAR-EPSCs increases starting from P6 and decreases around P13, when callosum development is almost complete. Using bilateral enucleation performed at different ages, they identified a critical time window (P4–P6) during which retinal influences induce processes that slow down the kinetics of NMDAR-EPSCs (Olavarria et al., 2007).

Following these evidences, it would be straightforward to think that NMDARs were necessary for callosal map establishment. This hypothesis was tested by performing a blockade of NMDARs with MK-801 during P4–P6. Surprisingly, NMDARs blockade did not produce massive callosal map deficits, ruling out the hypothesis that NMDARs were the unique players in determining callosal topography. On the other hand, some abnormalities were observed, like alterations in the total length of arbors, when NMDARs blockade was performed at P6. Since at P6 the callosal map is already established, NMDA regulation could be involved in the maintenance of the map, rather than in its establishment (Olavarría et al., 2008).

Very few studies analyzed functional consequences of enucleation (Toldi et al., 1989; Olavarria et al., 2007; Barb et al., 2011; Kozanian et al., 2015). One of these is from Toldi et al. (1989) in which they recorded rats into the lateral portion of the V1, in the cortex contralateral to the removed eye. Authors found that callosally evoked responses were larger than normal. Authors presented morphological data supporting the idea that the increased callosal responsiveness could be due to an high density of callosal terminals in layers II and III (Toldi et al., 1989).

Although studies on callosal reorganization following eye enucleation have contributed to shed light on the plasticity of interhemispheric connections in the visual cortex, a major confounding effect is the anterograde degeneration of optic fibers. Thus, influence of total removal of retinal activity has been studied also exploiting pharmacological blockade by TTX. Rearrangement of callosal connections has been studied in rabbits by repeated application of TTX starting on postnatal day 6–7. These animals displayed a widespread callosal zone, extending for one-third more into area 17 than in control animals (Murphy and Grigonis, 1988). Interestingly, the findings resemble those results obtained performing enucleation at birth (Grigonis and Murphy, 1991). If the TTX treatment was prolonged for the first two postnatal weeks, no deficits could be detected regarding the band of callosal terminals into the visual cortex ipsilateral to the untreated eye (Olavarria et al., 1987; Chang et al., 1995). Thus, pharmacological blockade of spontaneous retinal activity seems to be critical in a different time window with respect to eye removal.

### Eyelid Suture

A further approach for testing the role of input activity on CC organization consists in eyelid suture, which differs from retinal activity blockade because it spares spontaneous retinal activity, eliminating only patterned activity. Early studies with bilateral eye suture found a reduction of callosal cell number, but also extension of callosal zone into the contralateral cat visual cortex (Innocenti and Frost, 1979; Innocenti and Caminiti, 1980; Innocenti, 1981). The reduction of callosal neurons seems to be restricted to this experimental procedure (bilateral suture), because data coming from bilateral eye enucleation (see above) do not reduce significantly callosal neuron number, although it also causes a disorganization of the callosal zone.

Classically, the standard paradigm used to test how a patterned afferent activity could shape visual system is undoubtedly MD. This technique was first introduced by the pioneering experiments and seminal studies of (Wiesel and Hubel, 1963; for a review see Constantine-Paton, 2008) on visual plasticity in mammals. It is well established that MD performed during the critical period induces a weakening of deprived eye responses and loss of target regions, followed by strengthening of the open eye (Frenkel and Bear, 2004). These processes trigger a shift in OD of visual cortical neurons toward the open eye. The importance of this finding was immediately evident in view of a potential therapeutic treatment of children with amblyopia (i.e., lower visual acuity due to unbalanced input coming from both eyes; for a review see Levi and Li, 2009).

OD plasticity triggered by MD was in fact exploited as scientific rationale of patching the healthy eye of an affected child, to strengthen responses of the “lazy” eye and restore a correct vision. The effect of the treatment is proportionally inverse to the age of subjects. As a consequence, many researches are focused to find treatments for adult subjects. Since the long-term MD results in permanent loss of visual acuity (amblyopia; Morishita and Hensch, 2008), prolonged MD has become soon a classical model to study modifications triggered by compromised binocular inputs and a protocol in order to test therapeutic treatment, especially in the adult brain (Sale et al., 2007; Sengpiel, 2011; Spolidoro et al., 2011; Baroncelli et al., 2012; Tognini et al., 2012).

Regarding the CC, alternated MDs in cats during brain development produce anomalies in callosal projections, reducing number of callosal neurons in supragranular layers and expanding callosal zone at the border between areas 17 and 18 (Frost et al., 1990). Additional results were obtained in a recent article where monocularly deprived cats displayed high variability in the organization of OD columns within the callosal zone. Some eye columns showed ectopic callosal cells, probably due to an impaired elimination, triggered by experience during development (Alekseenko et al., 2009).

An interesting research article investigated in cat the effect on callosal map of MD performed in adulthood (Watroba et al., 2001). Authors applied a procedure in which they deprived one eye, 6 weeks later they sectioned the optic chiasm and 3 days later they recorded from V1. This protocol excludes long-term changes due to optic chiasm section itself. Anatomically, in monocularly deprived animals, they did not find any difference compared to normal cats, callosal units mainly resided at the 17/18 border, and the only detectable change in retinotopy was a wider callosal visual field towards the ipsilateral hemifield.

It is not surprising that manipulation of visual experience in adulthood does not produce a huge effect if compared with that obtained by manipulation during the critical period, but the take home innovative message found in this article was that the authors described functional changes in responsiveness of callosal units. Indeed, Watroba et al. (2001) reported abnormally large receptive fields and a loss of orientation selectivity, usually found during development. These data point out that even without evident anatomical changes and in adulthood, callosal inputs are able to respond and to contribute to cortical plasticity.

Our group was involved in a study on the functional changes in callosal input following MD, during the critical period (Restani et al., 2009). We used MD to induce the expected OD shift in cortical units. Then we analyzed OD distribution before and after an acute pharmacological removal of callosal input, applying muscimol into the cortex opposite to the recording site. Remarkably, this acute inactivation was able to partly reverse the effect of MD, shifting OD distribution again toward the contralateral, deprived eye. Data demonstrated that the OD recovery was due to a specific increase in the strength of the deprived eye. It is clear that such a fast recovery implicates functional rather than anatomical changes occurring during MD period. We suggested that acute removal of callosal input following MD unmasks deprived eye inputs. Thus, MD induces functional changes in interhemispheric output, inhibiting deprived eye responses. Thus we further tested the idea that callosal function is necessary for the occurrence of MD effects. We verified this hypothesis performing a continuous silencing of callosal input throughout the MD period. We prevented the loss of responsiveness of the deprived eye, and we found a dramatic reduction of the OD shift (Restani et al., 2009). Our data unveiled first of all, that callosal input could switch its role in physiological or pathological conditions, and, secondarily, that CC is a critical source of inhibition in visual cortical plasticity. Since about 99% of callosal neurons are excitatory neurons, it is likely that influence on inhibitory circuits takes place through disynaptic contacts on local inhibitory neurons in the target hemisphere (Toyama et al., 1974; Innocenti, 1980; Restani et al., 2009). It is interesting to mention that transcallosal inhibition appears to play an important role in plastic events occurring during others brain pathologies (see below).

It is also worth noting that MD induced a switch from interocular to interhemispheric suppression. Indeed, in control non-deprived young rats, intravitreal acute inactivation of the ipsilateral eye by TTX injection had no impact on contralateral eye VEP responses, whilst ipsilateral eye responses were greatly enhanced after injection of the contralateral eye with TTX. These data evidenced the existence of a suppressive mechanism by which contralateral eye activity inhibits responses coming from the ipsilateral eye (Pietrasanta et al., 2014). Remarkably, in MD rats, this mechanism was lost, because contralateral eye inactivation with TTX no longer unmasked ipsilateral eye responses after 7 days of eyelid suture. The authors investigated whether ipsilateral open eye acquired the ability to suppress weak responses from the contralateral, deprived eye, via thalamo-cortical pathway or callosal route. TTX injection into the ipsilateral eye failed to unmask contralateral eye responses, while on the contrary acute silencing of callosal communication by muscimol in the opposite hemisphere triggered an enhancement of contralateral eye VEPs (Restani et al., 2009; Pietrasanta et al., 2014). Thus, plasticity of callosal communication induced by eyelid suture represents a key step in the OD shift occurring during MD.

### Strabismus

Already Elberger et al. (1983) investigated callosal organization in optically induced strabismic cats. They found an enlargement of callosal zone, with somas of callosal neurons occupying a wider portion of visual cortex. The authors did not interpret this result as a failure in eliminating exuberant connections, but rather they argued that the wider area would reflect the necessity to increase the integration between the two hemispheres (Elberger et al., 1983). Similar expansion of callosal area was observed by other groups that reported a wider portion of the callosal zone in cats, after surgically-induces strabismus (Berman and Payne, 1983; Milleret and Houzel, 2001; Alekseenko et al., 2006, 2009). Thus, an increased callosal connectivity between cortical regions mapping peripheral portion of the visual field may ensure greater communication between the two hemispheres in strabismus. The wider distribution could be due to a decreased competition between inputs coming from the two eyes (Alekseenko et al., 2006). It is noteworthy that strabismus is effective in enlarging callosal zone even when induced as late as postnatal day 36 (Berman and Payne, 1983). However, contradictory results were also reported by other groups, that found no changes in size of callosal zone (Bourdet et al., 1996). These discrepancies could be due to different methodological experimental procedures and different onset of pathology.

Functionally, one of the first reports which addressed interhemispheric changes in human strabismus was made by Lepore’s group (Roy et al., 1994). Authors tested whether strabismus was able to produce deficits in the connectivity of the CC. They measured the interhemispheric transmission time (ITT) in strabismic and normal subjects. Despite the findings of a reduced speed of response in the central visual field in the amblyopic eye, authors did not measure significant differences for IIT in normal and strabismic subjects, with or without amblyopia (Roy et al., 1994), suggesting no evident impairments of callosal connectivity. On the other hand, electrophysiological data suggest callosal plasticity in strabismic cats and monkeys (Milleret and Houzel, 2001; Watroba et al., 2001). In strabismic cats, recordings from V1 showed that callosal neurons had decreased binocularity, and gained the ability to respond to fast-moving stimuli, two features typical of strabismus (Milleret and Houzel, 2001). In addition, strabismus reduces orientation selectivity of callosal neurons and enlarges their receptive fields, similarly to what happens following MD. In particular, in monkey and cats some units became responsive to stimuli when presented in the ipsilateral hemifield, although these units were not selective for orientation or motion (Sugita, 1996; Milleret and Houzel, 2001; Watroba et al., 2001). By combining retrograde tracing of callosal cells with imaging of OD, it was found that in strabismic cats, callosal connections link predominantly territories that share the same OD (Schmidt et al., 1997).

Recently, the impact of strabismus on development of callosal connections was anatomically investigated in cats. Reconstruction (3D) of single callosal axons shows that unilateral convergent strabismus triggers abnormalities in callosal connections (Bui Quoc et al., 2012). Specifically, strabismus induces an extension of interhemispheric terminals into the hemisphere ipsilateral to the deviated eye. This results in an asymmetrical interhemispheric connection through the CC, which in turn prevents the setting of a coherent representation of the two visual hemifields.

### Blind Subjects

Early post-natal blindness caused by bilateral peripheral damage (retinis pigmentosa, tumors such as retinoblastoma, glaucoma, accidents) may induce plasticity of the CC in humans. For example, Leporé et al. (2010) mapped the brain of blind adult subjects in search of differences in white matter structure. This study included subjects with early (<5 years old) or late (>14 years old) blindness onset and a similar number of matched controls. Using tensor-based morphometry, authors found a significant volume reduction in primary and secondary visual cortices in early onset subjects. Interestingly, however they measured an unpredicted decrease in volume also in other regions, like the premotor area. Other parts, such as frontal white matter, showed an increased volume, suggesting the activation of some compensatory mechanism. In the late onset group, differences in volume were less widespread, although still significant. In this case the highest reduction was measured in correspondence of the occipital regions. Specific reductions in callosal connectivity were found in the isthmus and splenium of the early onset group, while this did not occur in late onset subjects, possibly because development of CC is almost complete, reducing the impact of lack of visual input (Leporé et al., 2010). Coversely, subjects affected by early blindness got reduced input to visual cortex and CC during the phase of active myelination, which is, as a consequence, diminished. These interpretation fits well with the finding that neuronal activity promotes oligodendrogenesis and increases myelination (Gibson et al., 2014). Another study examined the CC in a cohort of congenitally blind individuals (Tomaiuolo et al., 2014). The authors found that the splenium was significant smaller in blind subject as compared to control. This observation is consistent with a study of resting state fMRI that reported decreased functional connectivity between the two occipital cortices in congenitally blind subjects (Liu et al., 2007). Butt et al. (2013) found an alteration of the pattern of between-hemispheric striate correlation, but age of blindness onset had no impact on this parameter.

A study by Bock et al. (2013) thoroughly examined volume, diffusivity and topographic organization of visual callosal connections in control subjects, in early blind (0–5 years old) patients and in a group with another extreme severe condition such as anophthalmia in young subjects. The condition of this latter group of subjects gave the authors the opportunity to expand the study, not confined to the impact of blindness on callosal organization, but specifically considering the effect of spontaneous retinal activity (in early blind, before they lost sight) or in absence of retinal input (not only lack of patterned vision). The analyses made by authors were able to confirm the retinotopical organization already reported in literature (Dougherty et al., 2005; Saenz and Fine, 2010). However, they failed to measure splenium volume differences in both early blind and in anophthalmic subjects. No differences were found in radial diffusivity or fractional anisotropy within the splenium of the CC among sighted-control group, anophthalmic or early blind subjects. Quite surprisingly, authors only detected reduced mean diffusivity and possibly reduced longitudinal diffusivity in anophthalmic subjects, but not in early blind subjects. This could reflect a reduced sensitivity of parameters such as diffusivity and anisotropy.

The study by Bock et al. (2013) also focused on how visual deprivation affects the topographical organization of fibers within the splenium itself. The topographic organization of visual callosal projections within the splenium in control-sighted was confirmed, however, here the novelty is represented by the findings that the coarse arrangement was not disrupted either in early blind or in anophthalmic subjects. Thus, they conclude that retinal activity does not play a key role in topographic outline of visual callosal axons within the splenium in humans.

Another study by Barb et al. (2011) has used DTI to investigate structural features of the CC in subjects with retinoblastoma, a childhood cancer of the eye. The authors found evidence for disruption of white matter maturation, in particular patients with bilateral retinoblastoma had greater deficits compared to those with unilateral disease. However, these abnormalities were transient and fractional anisotropy progressed towards normal values with the increase of age of the patients (Barb et al., 2011).

It is quite clear from these examples how, especially in humans, the study of the effects of manipulating retinal input on CC could be complex and challenging. Contradictory results from distinct research groups could certainly reflect the difference in methodology, but we also need to take into account a wide degree of variability and differences in white matter circuitry in the cortex of individual subjects (e.g., Putnam et al., 2010).

## Brain Damage, Corpus Callosum and Visual Cortex Responsiveness

It is unnecessary to remind that lesions in brain areas could be highly disabling. Motor impairments are often due to damage in frontal areas (Spalletti et al., 2014; Caleo, 2015; Gherardini et al., 2015; Lai et al., 2015). Cortical lesions could also occur in visual regions. Stroke in V1 or in the optic radiation lead to permanent loss of conscious vision (Grunda et al., 2013; Melnick et al., 2016).

Cortical blindness is a pathology in which, although the retina is functional, no visual input can be processed. When the lesion occurs in V1, which is a key hub for processing visual information, deficits involve all the aspects of vision: perception of color, motion, shape and many others. The scientific community is working hard to find and validate rehabilitating strategies, that might re-establish at least some aspects of visual abilities. For example, following damage to the V1, some patients exhibit “blindsight,” where they report a loss of awareness while retaining the ability to discriminate visual stimuli above chance. The pathways involved in this residual vision is controversial, with some studies attributing it to the retinotectal pathway via the superior colliculus whereas others implicate spared projections that originate predominantly from the LGN (Williams et al., 1995; Allen et al., 2014; Ajina et al., 2015). Functionality of these alternative pathways might be enhanced to circumvent deficits in visual cortex. On the other hand, cortical damage impacts also on integrity of cortico-cortical projections, and these could influence indirectly functionality of intact hemisphere. In patients with hemianopia, one half of the visual field is lost, but whether the “intact” hemifield is somehow impaired, is poorly understood. It is commonly held that the “intact” visual hemifield is perceptually unaffected, although some studies have challenged this point. For example, patients with unilateral occipital cortex injury show reduced spatial and temporal sensitivities in the sighted hemifield (Hess and Pointer, 1989; Rizzo and Robin, 1996). In addition, Paramei and Sabel (2008), underlined perceptual impairments in the “intact” visual field in hemianopes, specifically in detection of incomplete figures (Paramei and Sabel, 2008). Schadow et al. (2009) demonstrated deficits in visual processing of Gestalt patterns, because they found smaller amplitude and longer latency of gamma-band responses (which are associated with Gestalt perception processes) in hemianopic patients, in the unaffected hemisphere.

Deficits in contour integration and, consequently, in figure detection occurring in the “intact” hemifield of hemianopes might depend on dysfunction of interhemispheric connections coming from the damaged side (Restrepo et al., 2003). The issue that unbalanced interhemispheric input impacts on pathology or recovery of the contralateral hemisphere is supported by other human and animal studies (Moore et al., 1996; Restrepo et al., 2003; Corbetta et al., 2005; Fecteau et al., 2006; Fierro et al., 2006; Caleo et al., 2007). Moreover, based on their results, Paramei and Sabel (2008) argued that the interhemispheric input is likely to be involved in normal contour integration and shape segregation (Paramei and Sabel, 2008).

An interesting line of experiments investigated the potential therapeutic value of visual training. Visual stimuli were repetitively presented to cortical blind subjects in the blind visual quadrant, according to a protocol of visual training. This type of training was effective in enhance discrimination of some visual stimuli (Sahraie et al., 2006, 2010; Raninen et al., 2007; Huxlin et al., 2009; Das and Huxlin, 2010). However, the mechanisms by which training is able to elicit visual responses is not clear. In particular, further studies are needed to understand whether the recovery is due to the recruitment of some intact zone in the lesioned hemisphere, or whether the contralateral healthy visual cortex is the principal effector.

A recent study analyzed functional consequences of lesions in V1 subjects before training (Kavcic et al., 2015). Their goal was to evaluate which circuits were still functional and therefore available as a substrate for recovery. They recorded VEPs in control and cortically blind subjects with unilateral damage to V1 and/or optic radiation. The VEP components analyzed were amplitude and latency related both to stimulus-onset and motion-onset. In the damaged brain hemisphere, stimulus onset VEPs could only be recorded when the stimulus was presented in the unaffected visual field of blind subjects. This phenomenon implicates a transfer of visual information through callosal projection from the healthy hemisphere to the lesioned one. These findings demonstrate that in these cortically blind subjects the damaged cortex still has some functional area or undergoes plastic reorganization which allow to respond to visual stimuli, via interhemispheric pathways. Forced visual training could be able to enhance the function of callosal pathways and this might support recovery of simple visual performance also in the damaged hemisphere, obviously depending on the extent of the lesion (Figure 1B).

A deeper knowledge of spared circuits, including callosal projections, able to elicit VEPs in cortically blind patients is clearly necessary to optimize possible rehabilitating strategies. Previous reports have studied cortical responsiveness in cortically blind patients. In some cases, abnormal VEPs could be recorded from damaged V1, but in most of the cases no VEPs could be detected (Aldrich et al., 1987; Biersdorf et al., 1992; Watanabe et al., 2007). Two companion articles from Innocenti’s group analyzed deeply visual functions of two patients (MS, FJ) with bilateral lesion of the V1 (Kiper et al., 2002; Knyazeva et al., 2002). Although lesions were similar, they occurred in different developmental stages in the two children (gestational age 33 weeks in MS and at postnatal month 7 in FJ). Researchers performed neuropsychological, psychophysical and physiological studies, finding both similarities and differences in their conditions. Surprisingly, both patients displayed minimal deficits in basic visual functions, such as visual acuity, contrast sensitivity, color, form, motion perception. However, deficits became relevant when authors analyzed higher-order visual processes, like figure construction or figure-ground segregation. Interestingly, despite similar lesions, patient MS underwent a dramatic amelioration of impairments between 4.5 and 8 years (Kiper et al., 2002). To clarify the physiological mechanisms involved in the recovery, authors took advantage of fMRI to identify areas that were still functional (Knyazeva et al., 2002). In both patients, they found isolated islands of the V1 that could be still activated by visual stimulation. Interestingly, authors quantified also CC functionality using EEG coherence analysis. This analysis allows to measure in humans and animals the coherence of the two hemispheres (thus, functionality of CC) presenting iso-oriented or orthogonally- oriented gratings in the two hemifields, close or far from the vertical meridian (Kiper et al., 1999; Knyazeva and Innocenti, 2001). In normal subjects, interhemispheric coherence (ICoh) should increase when stimuli are presented close to the vertical meridian, and only with iso-oriented gratings, because, as we already discussed above, CC preferentially interconnects neurons selective for similar stimulus orientation (Schmidt et al., 1997, 2010; Makarov et al., 2008; Rochefort et al., 2009). Knyazeva et al. (2002) found in FJ, a patient whose recovery of visual perception was less than that in MS, no consistent changes in ICoh over the time. On the contrary, the patient MS displayed visual stimulus-dependent increase in ICoh starting at 7 years. These results speak in favor of the hypothesis that functional recovery in MS might be, at least in part, due to the functional maturation of her cortico-cortical connections, including callosal fibers. Functional recovery observed in MS seem correlated with the functional maturation of interhemispheric connections, thus these studies emphasize how transcallosal input could be a key factor in the recovery of function following cortical lesions, especially during an early developmental stage (Kiper et al., 2002).

## Contribution of Transcallosal Input Dysfunction to Brain Pathologies

We have reviewed numerous data showing how alterations in visual input or cortical areas could impact on callosal organization and function. On the other hand, anomalies in callosal input physiology could contribute to brain pathologies, such as it has been seen in “neglect”. Neglect patients do not pay attention to the left spatial hemifield, due to lesion in the right parieto-frontal cortex (Doricchi et al., 2008; Vuilleumier, 2013). Given evidence for transcallosal inhibition, researchers have exploited TMS in the unaffected side. Low-frequency rTMS induces inhibition of treated cortex, and a consequent improvement of performance in neglect patients (Oliveri et al., 2001; Fecteau et al., 2006; Fierro et al., 2006). Data from cats further support a role for callosum in neglect. Experiments showed that unilateral lesion of visuoparietal areas in cats produce a sort of neglect in the contralateral visual field (Payne et al., 2003). The authors performed cooling of the homotopic, contralesional areas, demonstrating recovery of attention to the neglected visual space.

Other pathologies could be, at first sight, independent of callosal input dysfunction, but following in–depth analysis a link has emerged. This is the case of photosensitive epilepsy (PSE), which is characterized by hyperexcitability of occipital cortical areas, leading to seizures following high contrast visual stimuli (like intermittent photic stimulation at high luminance and contrast; Covanis, 2005). A study from Porciatti et al. (2000) reported a loss of gain control in these patients. More specifically, in PSE subjects, at high contrast, VEPs responses were abnormally high in amplitude (Porciatti et al., 2000; Tsai et al., 2011). If we consider that, in healthy subjects, inhibition of one hemisphere by repetitive, low-frequency TMS unmasks visual responses at high contrast in the contralateral V1 (Bocci et al., 2011), one hypothesis is that callosal communication is impaired in photosensitive patients. Recently, a manuscript has addressed this issue (Figure 1C). The authors measured VEPs responses in drug-free patients with photosensitive seizures and healthy volunteers, before and following low frequency rTMS (Bocci et al., 2016). VEPs were recorded in both hemispheres before (T0), immediately after (T1) and 45′ following the completion of rTMS (T2). As expected, rTMS produced an inhibitory effect on VEPs amplitudes at all contrasts in the targeted side, while in the contralateral cortex facilitates responses at mid-high contrast in both groups, according to reports on removal of transcallosal inhibition (Restani et al., 2009; Bocci et al., 2011). The novelty of the study was to analyze differences between photosensitive patients and controls during the so-called recovery phase. VEPS responses remained persistently low at T2 in the treated hemisphere of control subjects, compared to PSE group, demonstrating a more prolonged inhibition in healthy subjects. The most exciting results were obtained in contralateral hemisphere. In PSE subjects (but not controls), the untreated side displayed a persistent enhancement of VEPs amplitude (Bocci et al., 2016). This suggests that an impaired callosal inhibition might be at the basis of this prolonged increase of visual responses in the untreated side, thus potentially contributing to the pathophysiology of PSE. Indeed, the most effective triggers for photo-paroxysmal responses are represented by high-contrast and mid temporal frequency stimuli (Porciatti et al., 2000; Fisher et al., 2005; Kasteleijn-Nolst Trenité et al., 2012), which are typically transferred across the callosal pathway (Berardi et al., 1987).

In addition, it is worth to cite a case study, LA, affected by congenital visual agnosia, caused not by visible lesions of visual cortex or optic radiation, but ascribable to a temporal-occipital epileptic focus (Knyazeva and Innocenti, 2001). Epileptic seizures were detected the first time at the age of 18 months in this patient. Although LA was subjected to therapeutic treatment, intermittent light stimulation was able to induce ictal seizures accompanied by blindness and bilateral posterior spikes on the EEG at around 12 years of age. Analyses of ICoh in this patient pointed out relevant differences compared to controls. Indeed in LA, ICoh increases equally when iso-oriented and orthogonally-oriented gratings are presented to the two hemifields, suggesting that the anatomo-functional setup for ICoh responses was altered. This means that callosal fibers had lost their property to link specifically iso-oriented neurons in the two hemispheres. Again, epilepsy occurring during development could influence connectivity, and finally this abnormal circuitry could contribute to the physiological phenotype of the patient (Knyazeva and Innocenti, 2001).

The literature discussed above underlies how callosal communication could be affected in visual cortical hyperxcitability, but cannot establish if this phenomenon is a consequence or if it concurs to the pathology. A study in animals addressed the effect of epileptic activity on development of callosal fibers (Grigonis and Murphy, 1994). Rabbit visual cortex was continuously injected with penicillin in postnatal age to induce epileptic activity. Callosal projections were anatomically analyzed in rabbits at 4 weeks and authors found that fibers in the penicillin-infused hemisphere were not restricted to the typical narrow zone at the border between area 17 and 18, but they retained a diffuse pattern, characteristic of neonatal development. Thus, epileptic activity could actively consolidate the immature callosal pattern, leading to the establishment of a diffuse callosal arborization. Although authors did not record physiologically from visual cortex, anatomical abnormalities might reflect physiological differences, which could contribute to the cortical impairments reported in patients following childhood epilepsy and to deficits in visual processing reported recently in one animal model of focal neocortical epilepsy (Vannini et al., 2016). Along this line, a recent article has reported an enhanced neuronal activity in the hemisphere contralateral to a lesion in rat visual cortex (Imbrosci et al., 2015).

## *Shedding Light* on Callosum: Optogenetic Dissection of Interhemispheric Visual Inputs

Up to now, we reviewed works that exploit non-invasive imaging techniques in humans, like TMS, to investigate physiology of callosal connections and examine changes following alterations of visual inputs. We have also discussed how callosum could be modified following brain lesions, and its possible involvement in specific pathologies, like neglect or occipital epilepsy. Overall this literature emphasizes the importance of understanding interhemispheric function in cortical processing and plasticity phenomena.

Since the past few years, progresses in neuroscience techniques such as two-photon imaging and optogenetics, have facilitated evaluation of neural circuits *in vivo* (Carter and de Lecea, 2011). In particular, optogenetics includes a variety of techniques by which neurons could be activated (or inhibited) by shedding light directly on the cells. The majority of experiments are designed so that light-dependent proteins are expressed in specific type of neurons (Fiala et al., 2010). This is the “dream of physiologists” come true: to be able to selectively switch on (or off) specific cell types, and analyze their contribution to specific circuit responses.

Such optogenetic techniques are ideal to study callosal pathways. Neuron activation could be easily performed in one hemisphere, exciting with light the neurons in this side of the brain, while in the contralateral hemisphere circuitry response could be analyzed by electrophysiological imaging techniques. One of the big advantages of this experimental design is represented by clear spatial separation between area of opto-activation and area of recordings, decreasing the possibilities of artifacts or multiple neuron type activation. Thus, is not surprising that callosal fibers have been target of optogenetic experiments, in particular those connecting somatosensory cortices (Petreanu et al., 2007).

In the field of visual system, one particular research regarding CC must be mentioned (Sato et al., 2014). Carandini’s group expressed Channelrhodopsin-2 (ChR2)-Venus in pyramidal neurons of one hemisphere. They got expression in layer 2/3, where a high percentage of callosal terminations is present, and they decided to stimulate by pulsed laser these terminations, in order to elicit antidromic potential to activate callosal neurons in binocular zone of the contralateral cortex. Light activation triggers spike activity in the contralateral cortex first in layer 2/3, but also in layer 5. Only spikes in deep layers were abolished by pharmacological blockade of glutamate receptors, suggesting that activity in superficial layers was due to antidromic activation. Interestingly, early activity was followed by a delayed and weaker activation of spiking in distal V1, in the monocular zone. They focused on this late activation in monocular area. They performed the antidromic optogenetic activation of callosal terminals concomitant to the presentation of visual stimuli in the monocular portion of the visual field. The authors found that neurons in the monocular zone showed clear activation in the absence of visual stimuli, but displayed suppression when their activity was elevated with a stimulus of 25% or 50% contrast. Overall, neurons showed a gradual transition from activation to suppression in the presence of visual stimuli of increasing contrast (Sato et al., 2014). Suppression was achieved only for activity recorded in the far monocular region, and only at high contrasts, while activation could be seen in both binocular and monocular areas.

Carandini’s group (Sato et al., 2016) further investigated this phenomenon. They used a similar experimental setting to address cellular mechanisms underlying distal modulation of intracortical activity (Sato et al., 2016). The authors performed patch-clamp recordings from the monocular zone, during 0% or 100% contrast stimulation. Recordings of membrane potential (*V*m) suggest that hyperpolarization induced at high visual contrast by callosal network activation might be a result of a decrease in excitation, instead of an increase in inhibition.

In any case, the functional effect of distal intracortical connectivity depends on the the level of feed-forward drive provided by visual stimulation. This might be the circuitry mechanism by which the CC acts as a gain controller in visual system (Wunderle et al., 2013; Bocci et al., 2016).

## Conclusions

Vision is a very important sensory ability in humans. Most research focused on the physiology and pathology of the eye, however it is clear that the integrity and function of all the elements of the visual system play an equally fundamental role. In this context, the CC connections provide a general and advantageous model for the study of cortical connectivity.

Here we reviewed how impairments of vision could affect morphology and physiology of CC. On the other hand, this induces plastic reorganizations which might in turn impact on visual activity and contribute to visual deficits. CC connections might become a possible target for future rehabilitation strategies, to help recovery of correct visual function and to counteract some brain pathologies.

New diagnostic and non-invasive intervention techniques have been developed, facilitating acquisition of new knowledge which may ultimately benefit patient treatments.

## Author Contributions

LR and MC wrote and discussed the manuscript.

## Funding

This work was funded by Fondazione Pisa (#158/2011), Ministero dell’Istruzione, dell’Università e della Ricerca (MIUR)-PRIN2012, Regione Toscana (RONDA project—Programma Attuativo Regionale cofinanziato dal FAS).

## Conflict of Interest Statement

The authors declare that the research was conducted in the absence of any commercial or financial relationships that could be construed as a potential conflict of interest.
